# Bernese periacetabular osteotomy (PAO): from its local inception to its worldwide adoption

**DOI:** 10.1186/s10195-023-00734-2

**Published:** 2023-11-02

**Authors:** Reinhold Ganz, Michael Leunig

**Affiliations:** 1 Faculty of Medicine, Dept. Orthopaedics, University of Berne, Murtenstrasse 11, 3008, Berne, Switzerland; 2 Department of Hip and Knee Surgery, Schulthess Clinic Zurich, Lengghalde 2, 8008 Zurich, Switzerland; 3 Eidgenössische Technische Hochschule (ETH) Zurich, Raemistrasse 101, 8000 Zurich, Switzerland

**Keywords:** Hip joint preservation surgery, Periacetabular osteotomy, PAO, Bernese PAO, Pelvic osteotomy history

## Abstract

The development of the Bernese periacetabular osteotomy (PAO) is based on a structured approach starting with an analysis of the preexisting procedures to improve the coverage of the femoral head and was followed by a list of additional goals and improvements. Cadaveric dissections with a detailed description of the vascular supply of acetabulum and periacetabular bone set the stage for an intrapelvic approach, which offered the largest acetabular correction possible combined with safe intracapsular access. The final composition of osteotomies required the development of several instruments and cutting devices before the feasibility could be tested on a series of cadaveric hips.

While the sequence of the osteotomies remained largely unchanged over time (except for the pubic and ischial osteotomies), several propositions for an easier/less invasive approach have been discussed; some made it into standard practice. Efforts were undertaken to optimize the learning curve and minimize failures using video-clips, hands-on courses, fellowships, publications, and ongoing mentoring programs. In retrospect, with almost 40 years of experience, such efforts have promoted a worldwide adoption of the Bernese periacetabular osteotomy.

## Introduction

Attempts to improve coverage of the femoral head date back to the beginning of last century, when Alessandro Codivilla, director of the famous Rizzoli Orthopedic Institute in Bologna, Italy, proposed a method to treat the established dislocation of the hip [1, 2]. He suggested to cover the femoral head completely by the joint capsule and to introduce it into a deepened acetabulum at the anatomic level. The technique was popularized 30 years later by Colonna and remains known under his name [3]. The procedure was executed until the late 1990s, when Boardman and Moseley in their follow-up paper concluded: “we do not support revival of this now obscure procedure” [4]. Nevertheless, better understanding of the blood supply to the hip region favored a revival of a modified version, now called capsular arthroplasty [5].

### Acetabular augmentation

Classic augmentation procedures of the deficient acetabulum are the shelf-arthroplasty [6, 7] and the Chiari osteotomy [8]. Like the capsular arthroplasty, they rely on transformation of the interposed capsular tissue to fibrocartilage. Compared with hyaline cartilage the mechanical quality of fibrocartilage is inferior, nevertheless, the survival of such a “neo-joint” can last up to several decades. The execution of the acetabular shelf augmentation is comparatively easy. Over time this technique underwent several minor modifications; it is still in use in France and in countries with French orthopedic influence [9].

The transverse juxtaarticular iliac osteotomy of Chiari is technically somewhat more demanding. It was first described in the early 1950s [8] and gained international acceptance for decades. Despite multiple articles reporting good long-term results [10–13], the procedure lost popularity with the extension of total hip replacement (THR) indication to younger patients and with the emergence of reorientation procedures.

### Acetabular reorientation

In these procedures the entire acetabular bone and hyaline cartilage is redirected over the femoral head; the gliding surface of the new articular relation with the femoral head remains all hyaline cartilage. Experience has proven that there is nearly always sufficient acetabular cartilage available to allow correct reorientation. LeCoeur was the first to execute such an osteotomy in 1939, but did not publish it before 1965 [14]. Today, several are of historical value, the majority is used in the country of the inventor, and few have reached international acceptance.

#### Single osteotomies

The Salter osteotomy is the only one wherewith a transverse and complete single osteotomy of the supraacetabular ilium is used to reorient the acetabulum[14]. It is preferred by pediatric orthopedic surgeons for its rather easy execution. However, with its pivot point near the pubic symphysis, correction is limited and interlinked with side corrections such as lateral, anterior, and caudad shift of the joint. The highest clinical relevance, however, has the tendency to create retroversion of the acetabulum, which in hips with low or no anteversion may create impingement problems in early adulthood. The Pemberton [15] and Dega [16] osteotomies are incomplete supraacetabular osteotomies correcting the roof only. The cut is similar as for a Salter osteotomy [14] but does not separate the ilium completely. While the Pemberton cut is straight, the Dega cut is curved following the acetabular roof. The deeper the cut penetrates, the easier the acetabular part of the bone can be bend downwards, decreasing the enlarged acetabular radius. Both techniques are used almost exclusively in premature hips with a shallow acetabulum. Both are technically more demanding compared with the Salter osteotomy [17]. Comparing long-term results, the Pemberton osteotomy seems to create slightly better results compared with the Dega and Salter osteotomies [18, 19].

#### Double osteotomies

Two double osteotomies for acetabular correction with different estimates have been described from Sutherland and Greenfield [20] and from Hopf [21]. The first is cutting through the bone near the symphysis, the second is cutting through the empty distal part of the paleo-acetabulum in cases of severe subluxation and secondary acetabulum.

#### Triple osteotomies

They can be divided into different types: those executed at a distance from the joint, which implies that the sacrospinal and sacrotuberal ligaments insert at the acetabular fragment and therefore limit the correction, and those executed close to the joint, which implies the ligaments are not attached to the acetabular fragment and therefore allow a higher spatial correction [14, 22–24].

#### Spherical osteotomies

These are performed very close to the joint, allowing extensive correction except for medial shifting of the acetabular fragment. The execution is rather demanding, and due to the acetabular vicinity of the osseous cuts, the fragment perfusion is limited to the obturator and capsular vessels. Simultaneous arthrotomy is therefore limited [25–27].

## The Bernese periacetabular osteotomy (PAO)

The most recently introduced reorientation procedure is the PAO [28]. In contrast to previous osteotomies, it is executed from anterior and from the inside of the pelvis. The procedure is the result of a research project with analysis of the limitations of the existing procedures, followed by extensive cadaveric dissections of the vascular supply and by trial osteotomies for best direction of the osteotomy cuts. Finally, the definitive version of the procedure was tested on 25 cadaver hips with the focus to test a set of new instruments.

Traditionally, pelvic surgery performed by orthopedic surgeons approached the acetabular area almost exclusively from the lateral side. With the progress in surgical treatment of trauma to the acetabulum and pelvis in the late 1970s and early 1980s by Letournel [29], as well as with better understanding of the vascular supply of the pelvis, the inner side of the pelvis became an interesting new surgical approach area. Additionally, known limitations of the preexisting augmentation and reorientation procedures (Table 1 [14, 15, 20–28]) raised the ambition for a more versatile procedure of acetabular reorientation that should fulfill several goals (Table 2).

**Table 1:** Characteristics of acetabular reorientation procedures

Study	Type	Distance to acetabulum	Limitation of correction	Physis crossing
Salter [15]	Single	Distant	+ + + +	No
Sutherland [20]	Double	Distant	+ + +	No
Hopf [21]	Double	Intraarticular	+ + +	No
LeCoeur [14]	Triple	Distant	+ +	No
Steel [22]	Triple	Distant	+ +	No
Tönnis [23]	Triple	Close	( +)	No
Carlioz [24]	Triple	Close	+ ( +)	No
Eppright [25]	Spherical	Very close	+	Yes
Wagner [26]	Spherical	Very close	+	Yes
Ninomiya [27]	Spherical	Very close	+	Yes
Ganz [28]	Periacetabular	Close	( +)	Yes

**Table 2:** Criteria to meet for a new reorientation procedure

All osteotomies from anterior and the inner side of the pelvis (preserved acetabular blood supply, unlimited intracapsular surgery, unaltered abductors)
Intact posterior column (hemipelvis stable, sacropelvic ligaments excluded, birth canal unchanged, sciatic nerve protected)
Patient supine, one approach (time saving, position optimal for intraoperative fluoro and control x-ray)

**Fig. 1:**
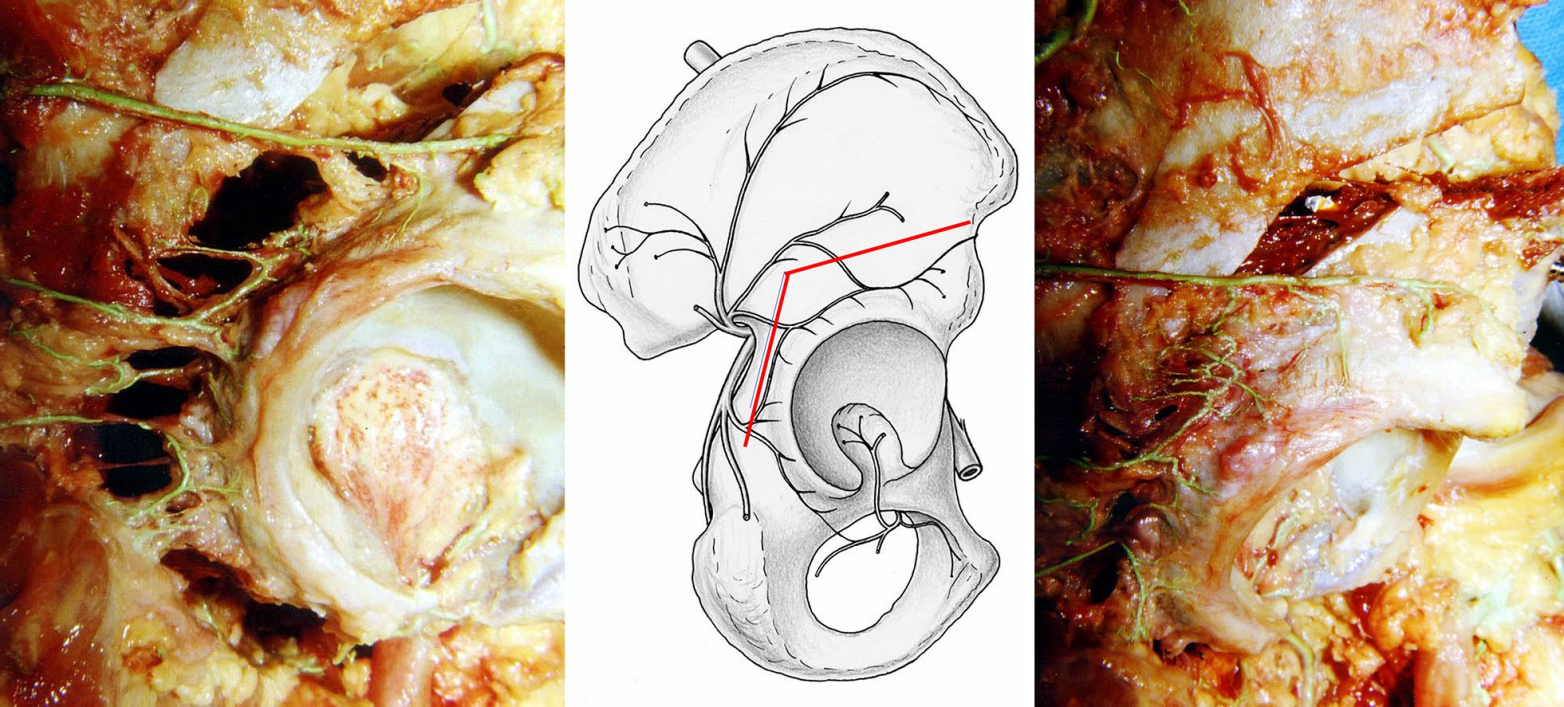
Periacetabular vascular supply cadaver study on vascular supply of the periacetabular bone. Right hip, osteotomies from pelvic inside. The branches from superior and inferior gluteal artery can be preserved during osteotomy (left) and fragment correction (right)

**Fig. 2:**
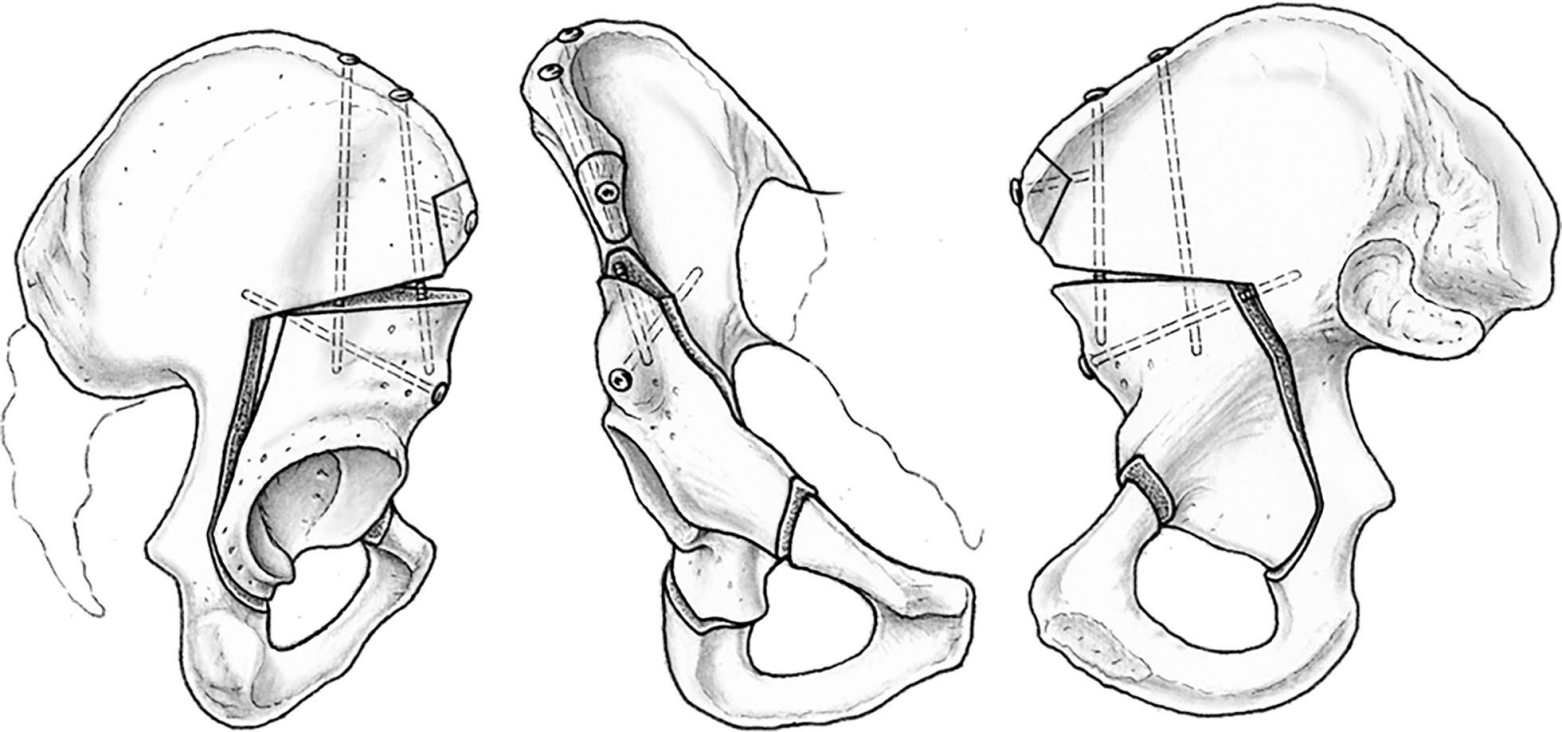
PAO osteotomy cuts arrangement of the five osteotomy cuts, visible on the left lateral view, in the middle anterior view, and on the right medial view of a right hemipelvis, namely incomplete ischial cut (first), pubis cut (second), supraacetabular cut (third), retroacetabular cut (fourth), and infraacetabular cut completing the ischial cut (fifth)

**Fig. 3:**
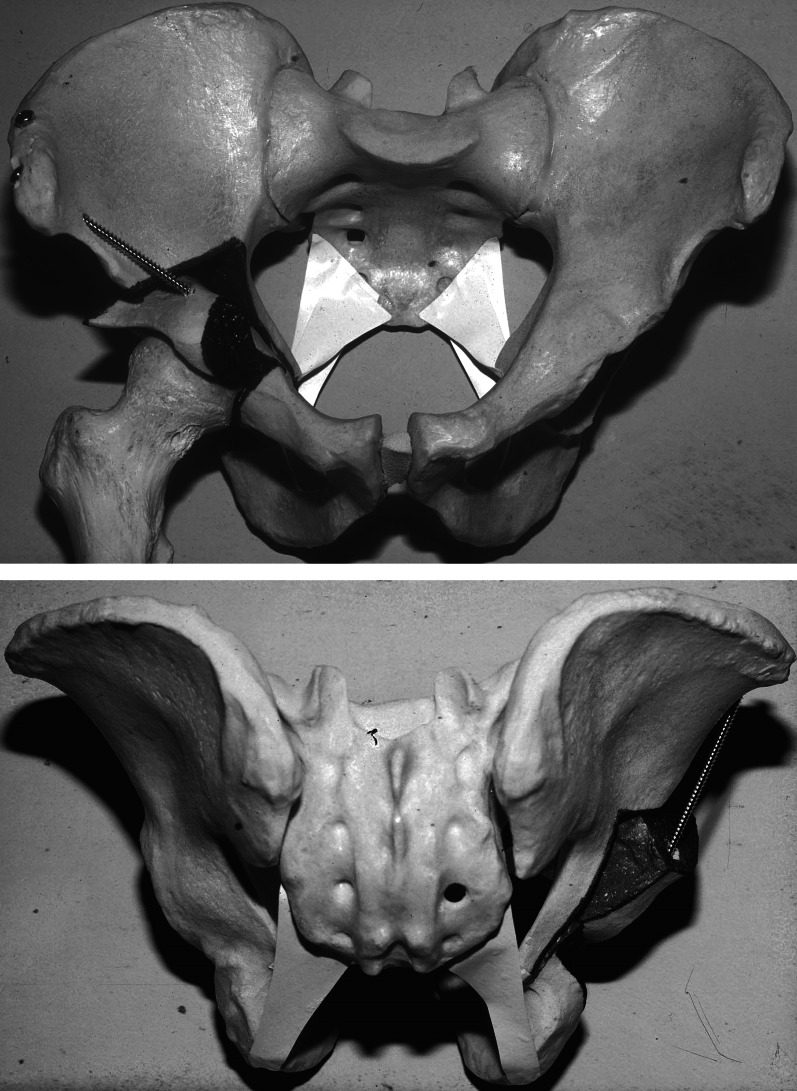
Pelvis plastic model: plastic model showing separation of the acetabular fragment from posterior column including sacrospinal and sacrotuberal ligaments. Top: anterior view; bottom: posterior view

**Fig. 4:**
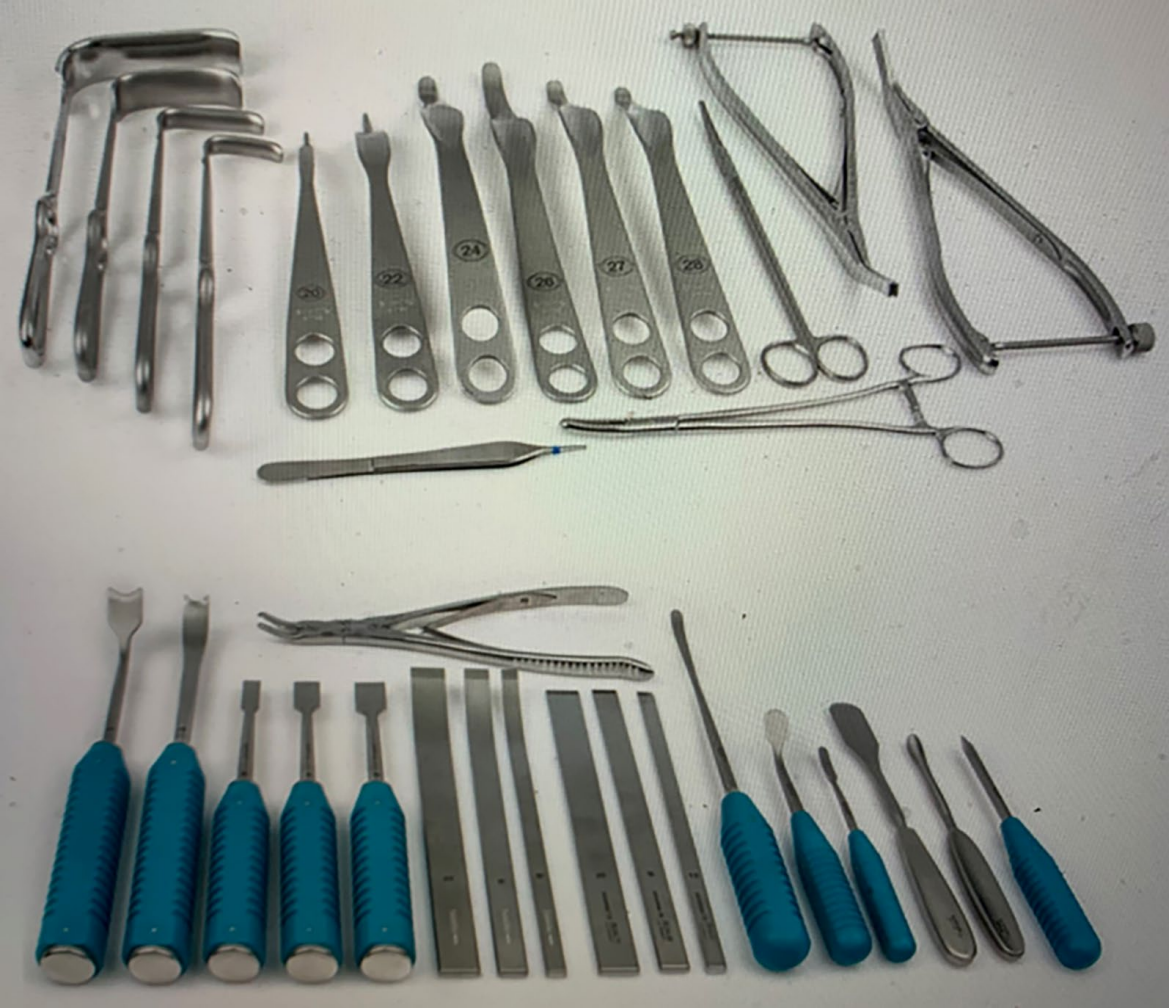
PAO instrumentation set of instruments with special osteotomes and retractors

The first step of the project was a cadaveric study of the vascular topography to the pelvis relevant to periacetabular osteotomies. Here it became obvious that the most crucial aspect was the preservation of the fragment vascularity during the osteotomies and during widening the gaps required for correction. It was best achieved if osteotomies were performed from the inner side of the pelvis [28] (Fig. 1). After first clinical introduction of the PAO, intraoperative laser Doppler flowmetry of the acetabular fragment was used and demonstrated that the signals drop during the reorientation but return to normal values after several minutes [30]. Overall, these tests took up 1 year, mainly due to limited availability of suitable cadavers. For the final sequence and orientation of the five necessary osteotomy steps, Jeffrey Mast, a clinical fellow from the USA at the time, was an ingenious contributor to the development of the PAO (Figs. 2, 3). Finally, we practiced on several cadaver hips to become familiar with technique and a new set of instruments, which later became commercially available (Fig. 4) from several providers.

**Fig. 5:**
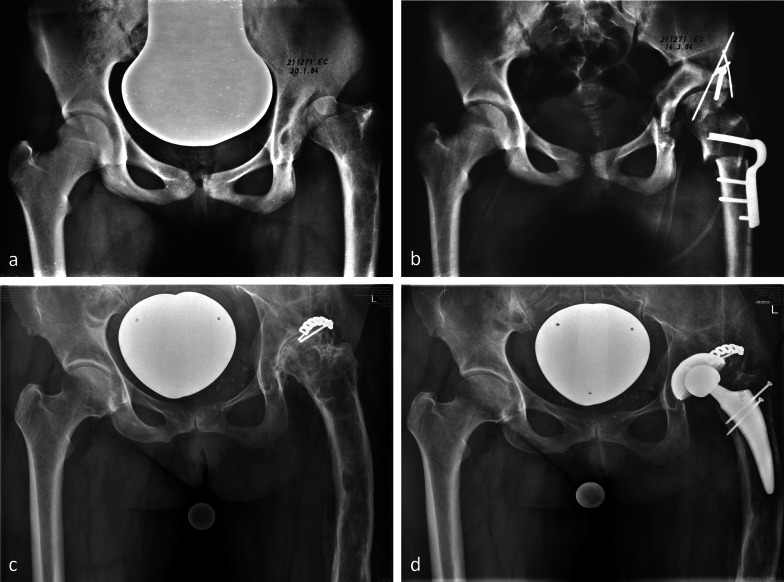
1984: first operated case—13-year-old female with PFFD of the left hip. **a** Varus proximal femur after intertrochanteric osteotomy 3 years earlier. **b** Periacetabular osteotomy followed by intertrochanteric revalgisation for better joint congruity. Good lateral coverage of the head. Increased retroversion of the acetabulum led to posterior subluxation 3 months after surgery, which was treated with a posterior shelf. **c** Progressive joint degeneration after 36 years. Plate for shelf fixation still in place. **d** Follow-up radiography of THR 2 years later. The patient regained a normal gait pattern

The first hip was operated on on 13 March 1984. We were waiting several weeks for an “easy” case, but Dr Mast could no longer postpone his flight back to the USA; this was the reason why we decided to treat the most urgent case on the list, which was a proximal femoral focal deficiency (PFFD) hip with complex deformity. The surgery of this 13-year-old girl took 4 h and 10 min and included a femoral valgus osteotomy to compensate for an earlier varus osteotomy. A total of 3 months after surgery, the hip dislocated posteriorly, a complication that was successfully treated with a posterior shelf. At that time, we did not know that PFFD hips have severe acetabular retroversion as a characteristic part of the malformation. The hip functioned well during 33 years before THR became necessary (Fig. 5). The results of the first 75 hips with rather heterogeneous forms of dysplasia, multiple precedent surgeries, and osteoarthrosis stage including Tönnis grade 2 were published 4 years later [28]; the success rate of this group dropped from 80% after 10 years to 60% after 20 years and to 30% after 30 years [31, 32]. Meanwhile, more homogeneous groups of hip dysplasia, younger age at surgery, exclusion of advanced osteoarthritis, as well as a concurrent arthrotomy for femoral head–neck shaping, have led to further increase of survival [33, 34].

## Technique

The patient is in supine position, the leg to be operated on is draped mobile. General anesthesia is standard as is blood salvage (cell saver). The approach is a modification of Smith Petersen’s primary description [35]. The sequence of the five partial cuts is described elsewhere in detail [28]. Some perform the pubic cut prior to the first ischial cut, especially when a rectus sparing approach is used. The classic exposure starts with osteotomy of the insertion of the sartorius origin at the anterior superior iliac spine (ASIS). Mainly in younger patients, the sartorius insertion including the inguinal ligament are released from the ASIS without an osteotomy. By incising the fascia of the tensor fasciae latae and moving away the muscle to the lateral side, the joint capsule is reached. By dissecting the iliacus subperiosteally, the medial flap can be mobilized. In the classic approach the reflected and the direct head of the rectus femoris muscle are divided and the iliocapsularis muscle is dissected from the capsule. Following the anteroinferior capsule to the inferior, the gap between capsule and psoas tendon further down to the obturator externus muscle is widened to reach the ischium. The deep branch of the medial femoral circumflex artery (MFCA) runs on the caudad surface of this muscle, which needs to be respected. Initially, for an incomplete cut of the ischium at the infracotyloid axilla, a specially angulated osteotome is used. Position and propagation can be controlled with fluoroscopy. In the rectus sparing approach, the ischium is approached medial to the rectus femoris, which is not released. The deep fascia is incised, and the ischial osteotomy is performed quite similar to the classic technique. In comparison with the classic technique taking down the insertion of the rectus, the rectus sparing approach is not really internervous, because the first branch of the femoral nerve is crossing the approach distally. The pubic cut, in the classic version second, is a complete separation of the pubis after subperiosteal dissection; the obturator neurovascular bundle is protected using two blunt retractors. The third cut is a supraacetabular horizontal separation of the iliac bone, starting just distal to the sartorius muscle origin. The cut is performed from the anterior and inner side of the ilium with an oscillating saw. During this osteotomy the abductor musculature is protected with a blunt retractor. The cut stops about 10 mm before the pelvic brim. For the fourth cut, a retroacetabular cut starts from there and is directed downwards at an angle of 110–120° posterior to the supraacetabular cut. Again, fluoroscopy helps to correctly propagate this cut using a 10 mm osteotome. At the angulation between the third and fourth cut, a curved osteotome is used to cut the outside cortex. At this point a bone spreader is inserted just distal to the angulation to open up the gap. With a retractor held against the quadrilateral surface near the ischial spine, the area for the fifth osteotomy can be opened. The cut is executed with a special osteotome and cuts the final osseous bridge between the fourth and the first cut. It separates the sacropelvic ligaments from the acetabular fragment (Fig. 3). When all cuts are properly accomplished, the acetabular fragment can be separated with a counter-rotating move of the spreader and a Schanz screw inserted into the iliac part of the acetabular fragment. The desired correction is temporarily fixed with two (−3) Kirschner wires and is controlled with fluoroscopy or a standard anteroposterior radiograph of the entire pelvis. The latter is preferred because it also guarantees the necessary orthograde position of the pelvis.

## Modifications of the approach over time

**Fig. 6:**
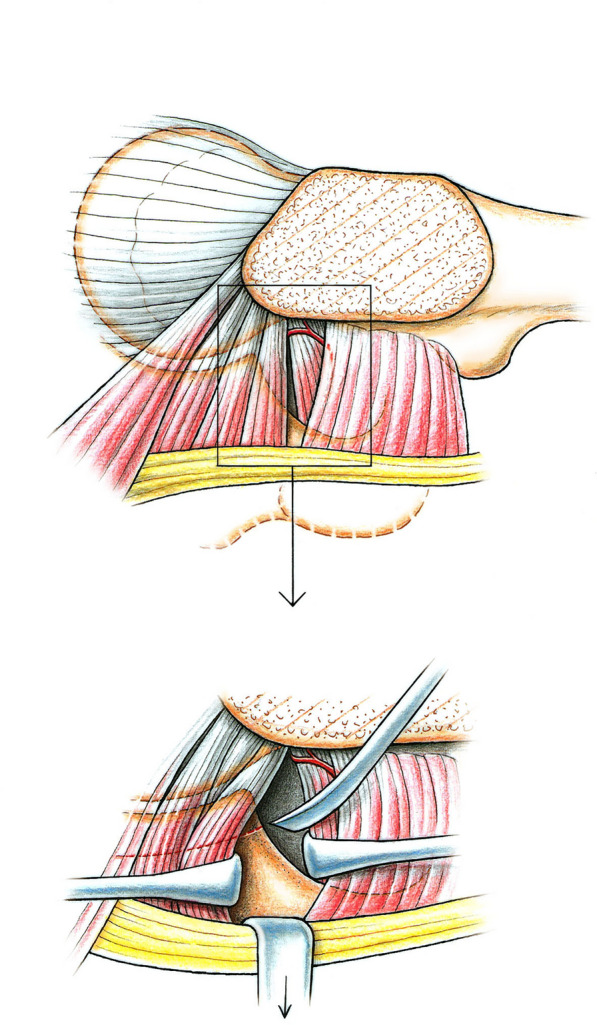
Ischial cut via femoral approach in hips with combined acetabular and femoral surgery. On top, access to the infracotyloid groove between obturator internus and gemellus inferior muscles. Bottom: it allows optimal protection of the ischial nerve during execution of the cut

Soon after the initial experience with 75 PAOs, the dissection of the abductor musculature was abandoned and sufficient protection of these muscles during the third cut could be realized with tunneling and placement of a blunt retractor [36]. An important insight came from follow-up studies, showing that some hips developed impingement symptoms after a correctly oriented acetabulum. A closer look revealed that concomitant femoral head–neck deformity is relatively frequent and was not producing symptoms before the PAO-improved anterior coverage. The problem can be detected by assessing internal rotation in hip flexion during surgery and solved with additional arthrotomy for osteochondroplasty of the anterior head–neck junction. Some authors recommend arthroscopy before PAO [34], which may compensate for missing or inadequate magnetic resonance imaging (MRI) and may even influence the indication for PAO. In complex deformities necessitating the combination of a PAO with a femoral procedure we are inclined to start with the femoral procedure and approach the infracotyloid axilla between muscles. gemellus inferior and obturator externus. For better discrimination it is advantageous to expose this gap before the femoral procedure; it allows to perform the first ischial cut under direct view with the sciatic nerve being held aside [37] (Fig. 6).

A more recent modification of the original technique is the rectus sparing approach, which allows the first cut to be performed by approaching the ischium medial to the intact rectus tendons. A cadaveric study describes the feasibility of this modification; however, intraarticular inspection and revision of the anterior inferior iliac spine (AIIS) might be somewhat restricted and the most proximal branch of the femoral nerve can be overstretched [38].

A number of proposals deal with modifications of the approach without changing sequence and configuration of the osteotomy. The attempts to execute the PAO using mini-invasive techniques, including the transsartorious approach [39, 40], may be applicable for minimal corrections but have limitations with complex deformities; consequently the widespread use remains limited. Another proposition is the use of a modified Stoppa or a pararectus approaches; both have been successfully performed for anterior acetabular fractures [41]; however, the usefulness for the execution of a PAO has not yet been demonstrated. An inguinal extension of the approach may increase the view on the pubic osteotomy area, but was nearly completely abandoned after several cases had postoperative deep vein thromboses (DVTs) of the femoral vein. Few are using a two-incision technique [42], similar to the Tönnis triple osteotomy [24]. A primary conception of the original technique was to avoid fluoroscopy and use only local landmarks for the execution. However, most surgeons today use standardized fluoroscopy control for some or even all osteotomies. Computer assistance has been proposed several times, but like for THR, so far it has not found its way into general practice. A most recent version [43] confirms that it may be helpful for the less experienced surgeon, a conclusion we gained already more than 25 years ago using a similar approach; it was given up after a short clinical testing period [44].

## Spectrum of the procedure

The following selected cases may demonstrate the spectrum of indication and therefore versatility of the PAO. While the procedure was originally intended as a method for primary acetabular dysplasia from developmental dislocation (DDH), it became useful soon after as an additional surgery for other complex deformities with acetabular participation, mainly Perthes or Perthes-like disease, but also in cases with femoral exostosis or septic arthritis with femoral head necrosis in the rare epiphyseal dysplasia and occasionally in early stage of protrusion and posttraumatic periacetabular deformities. Special challenge was constituted in cases after previous surgery, such as retroversion after Salter osteotomy or when a re-PAO was necessary.

### Case 1

**Fig. 7:**
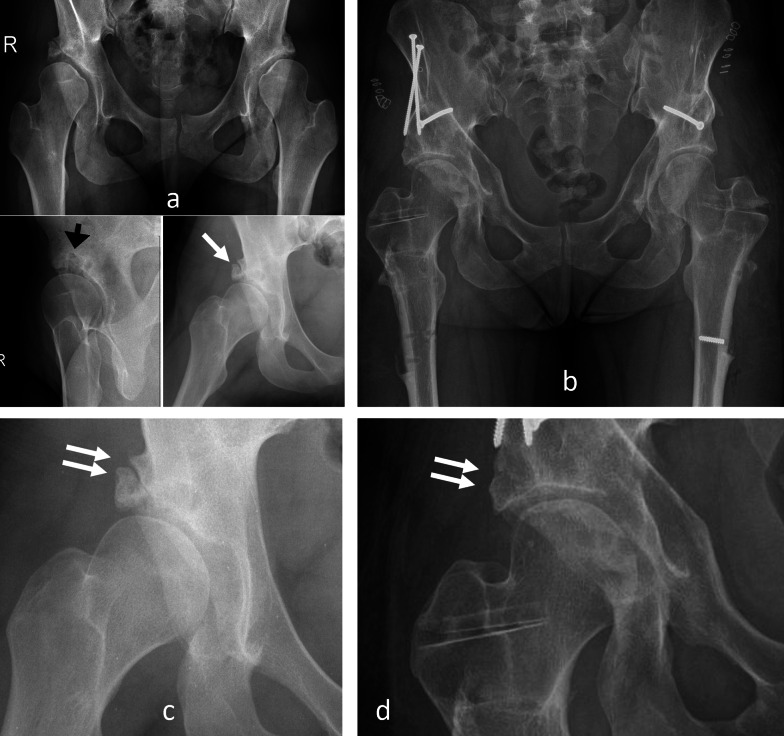
Bilateral severe acetabular dysplasia of 16-year-old female; more pain on the right side. **a** Several subchondral bone cysts, best visible in a lateral view (black arrow). Displaced rim fracture with some reduction in abduction (white arrow). **b** PAO combined with femoral varus osteotomy. A 6-month interval between surgery of the hips was observed (right side first). Slight overcorrection of the PAO on the right side for better unloading of the damaged rim area was performed. Partial metal removal after 2 years was performed. Radiological result observed after 5 years. **c** Closer look to the critical joint area of the right hip before surgery and after sugery (**d**) showing reduced and healed rim fragment (double arrow)

A 16-year-old female suffering from severe bilateral acetabular dysplasia and coxa valga with high fovea capitis had more pain on the right side. There was bilateral subluxation with fatigue fracture of the lateral acetabular rim on the right side (Fig. 7). The lateral view of the right hip showed substantial intraosseous ganglion formation in the roof, while the abduction view featured recentering of the femoral head and some reduction of the acetabular fragment. The complex deformity on both hips made it necessary to combine a PAO with a femoral varus osteotomy. Surgery started on the femoral level in lateral decubitus. Before executing the intertrochanteric osteotomy, the first ischial cut of the PAO was performed through this approach under direct view. For the remaining PAO cuts, the patient was turned into supine position. The acetabular rim fracture reduced spontaneously and did not need additional fixation. The left hip was operated on 6 months later. Healing of the osteotomies, including the rim fracture, was uneventful. Pain of both hips disappeared shortly after surgery, allowing for unrestricted age-appropriate activities. The prominent bend of the femoral implant created some discomfort, and this was the reason why partial metal removal was executed 2 years after surgery. A follow-up radiography at 5-year follow-up showed a perfectly reduced and healed rim fragment and disappearing acetabular cysts.

### Case 2

**Fig. 8:**
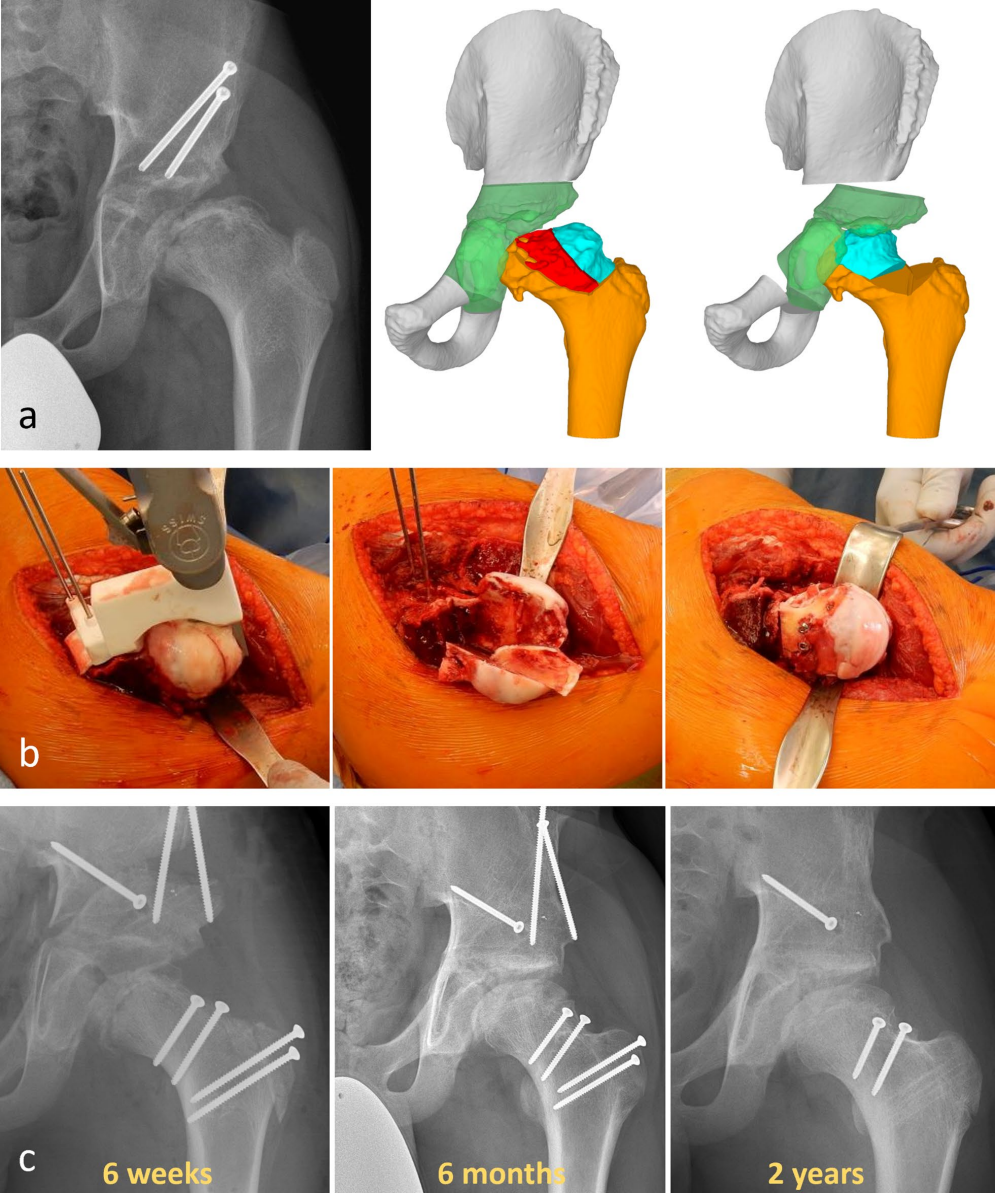
Perthes disease in the right hip of a 13-year-old male, operated on with Salter osteotomy. **a** Persistent subluxation and adduction with extrusion of the healthy lateral pillar and loading of the necrotic area. Adaptive widening of the acetabular cavity. On the right, computer simulation showing relocated head after resection of the necrotic area (red portion) and optimal coverage with PAO. **b** Intraoperative pictures showing the resection of the necrotic central part of the head with the template in place and the final size of the head after screw fixation of the mobile lateral part of the head with two screws. **c** Postoperative sequences with the new head healed without necrosis

A 13-year-old male with Perthes disease (Fig. 8). After Salter osteotomy pain did not subside and limping increased; both could be explained with persistent subluxation, widened acetabulum, followed by adduction contracture. Computer simulation showed reasonable reduction of the subluxated femoral head with intracapital osteotomy [45] and PAO. The precise execution of the femoral head osteotomy was facilitated by a cutting template prefabricated on the basis of the computer simulation data. Healing of the osteotomies was uneventful. Pain subsided completely; abductor training helped to regain a walking pattern without limping. At 1 year after surgery, partial metal removal helped to settle pain from projecting screw heads. At 2 years after surgery the configuration of the hip was close to normal, range of motion (ROM) was slightly less compared with the opposite side and function was unrestricted.

### Case 3

**Fig. 9:**
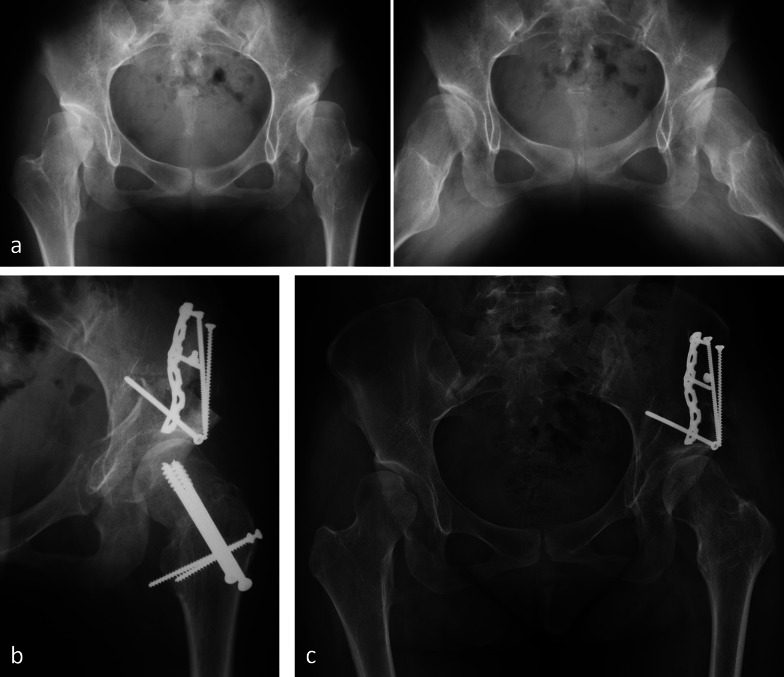
Acetabular dysplasia, multiple exostoses, and subluxation multiple exostoses near both hips with acetabular dysplasia in a very gracile 21-year-old female. **a** Pain and discomfort of the left hip during subluxation, palpable with rotation in full extension. Lateral view (right) showing the causative exostosis at the posterior neck. **b** Postoperative result after surgical dislocation, femoral neck varus osteotomy, and removal of the exostosis through the neck osteotomy, followed by PAO. **c** The 10-year-result. Removal of the femoral screws for local pain soon after surgery. Both hips are pain-free

A 21-year-old very gracile female with multiple exostoses around both hips and concomitant acetabular dysplasia (Fig. 9). She only had pain on the left side and described it as sudden, happening frequently during external rotation in full extension. The phenomenon could be demonstrated by ultrasound as impingement between a posteromedial neck exostosis and the infracotyloid ischium; the contact was followed by femoral head subluxation. Ultrasound also revealed that the causative tumor was bigger than its radiographic appearance. Both hips showed a high coxa valga and acetabular dysplasia. The surgical plan was to remove the neck tumor, correct the high neck valgus with a varus osteotomy, and to improve acetabular coverage with a PAO. Preoperative planning brought out that intertrochanteric osteotomy would not sufficiently increase the pelvifemoral clearance. On the other side, the risk of damage to the femoral head vascular supply was estimated to be high during an attempt to excise the big tumor via anterior and/or posterior retinacular dissection. Based on the knowledge that the posterior neck is free from blood vessels, it was decided to execute the resection through the neck osteotomy, which could also be used to perform the planned varus osteotomy. Again, surgery started with dislocation of the hip. Subperiosteal anterior and posterior dissection of the neck, containing the vessels to the head, was performed toward the base of the exostosis. It was followed by a mediocervical osteotomy and widening of the gap using a spreader. The view was sufficient to allow subperiosteal piece mill resection of the tumor under constant observation of bleeding of the head–neck fragment. Varus position of the neck was stabilized with two screws. PAO was executed as described earlier. Subluxation of the hip disappeared immediately after surgery. The 10-year result shows a reasonable hip joint with a wide joint space. The patient is pain free and the hip has a normal ROM. She is seen regularly by her local doctor who confirmed that the other hip is still functioning well.

### Case 4

**Fig. 10:**
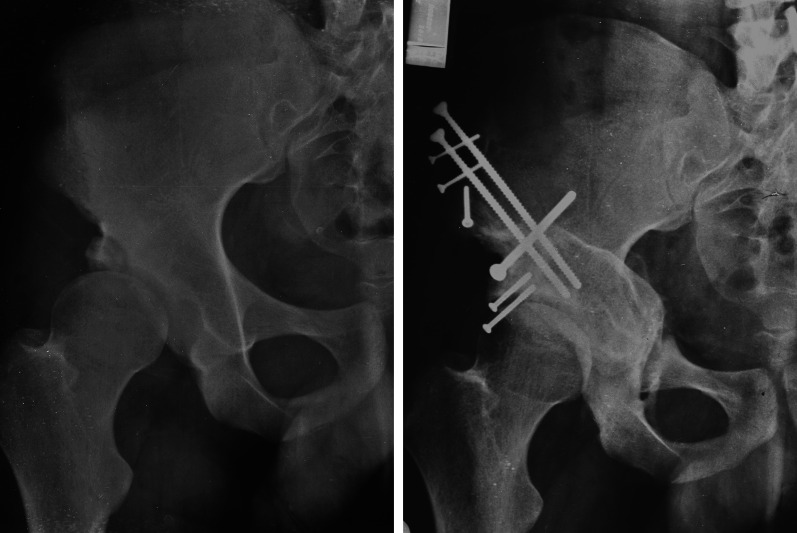
Posttraumatic dysplasia in a 19-year-old male. **a** Typical posttraumatic dysplasia of the right hip after a car accident at age 3. Large acetabular rim fragment. Laborious execution of the osteotomy due to the thick supra- and retroacetabular bone. Screw fixation of the large rim fragment. PAO with maximal possible medial displacement of the acetabular fragment. **b** Radiological result after 2 years with healed fracture and osteotomies

A 19-year-old rugby player with pain of the right hip, rendering him unable to participate in his favored sport (Fig. 10). History revealed that he was run over by a truck when he was 3 years old. He survived multiple fractures including a crush injury to the pelvic ring, all treated conservatively. After full recovery he had no further control until recently when he experienced increasing pain in the right hip during his sportive activities. Radiography revealed a typical posttraumatic dysplasia, apparently a consequence of the crush injuring the triradiate cartilage. This rare type of dysplasia is characterized by thickening of the inner wall, deformity of the hemipelvis, and retrotorsion of the acetabulum. Lateral and caudad growth of the physis leads to lengthening of the leg. The large acetabular fragment in this case is a consequence of chronic overload of the rim area. The surgical plan was to combine the acetabular reorientation with exceptional medial shifting of the lateralized acetabular fragment. Concerns about sufficient unloading of the fragment influenced the decision to fix it separately with two screws. The supra- and retroacetabular osteotomy cuts were laborious due to the unusually large diameter of the supra- and retroacetabular bone. Optimal medialization of the acetabulum led to minimal bony contact between acetabulum and hemipelvis, which was the reason why the correction of version was less than desired. Nevertheless, consolidation was uneventful. The patient resumed his rugby activities after 9 months, but although pain free, did not regain the necessary aggressivity for this type of sport and therefore gave it up. The 2-year result showed perfect consolidation of osteotomy and rim fragment with a large and congruent joint space.

### Case 5

**Fig.11:**
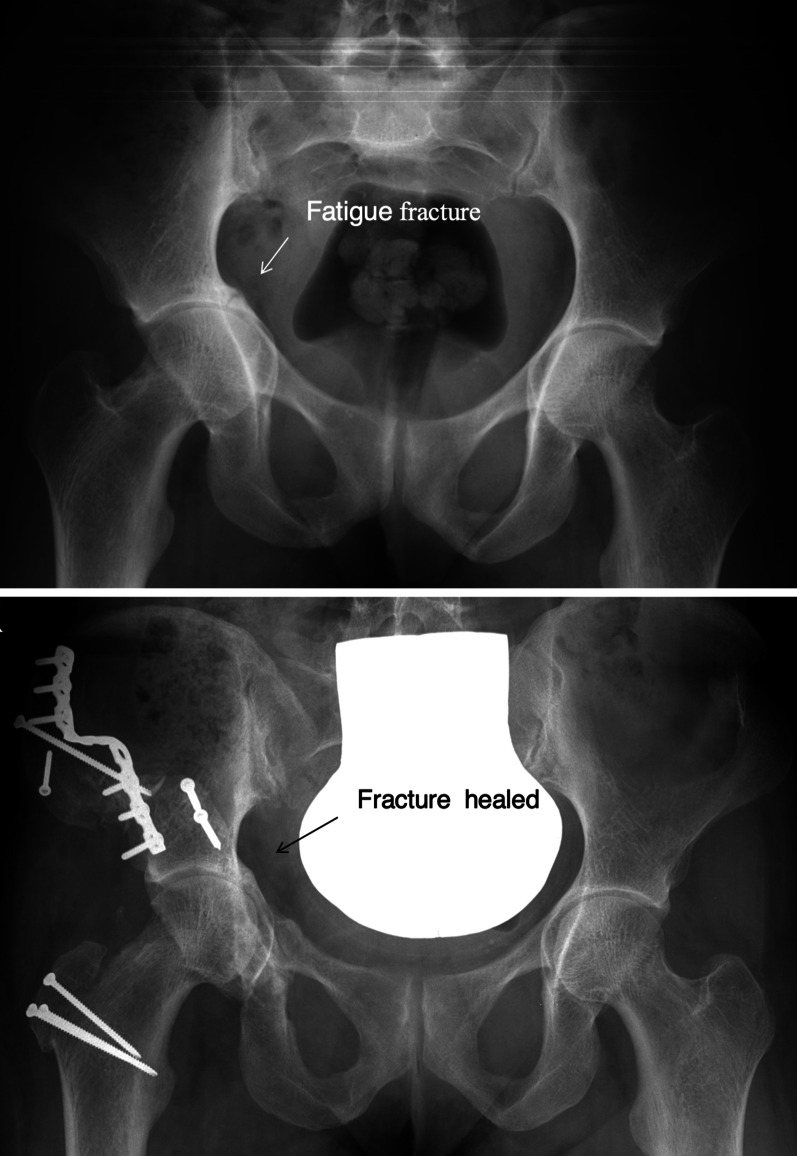
Acetabular protrusio was observed, with **a** bilateral protrusion in a 21-year-old lady with osteogenesis imperfecta. Constant pain on the right side while the left side was pain-free during most daily activities. Patient refused to get THR. On the top, preoperative radiography showing fatigue fracture through the bottom of the joint (white arrow). **b** Result 1 year after excessive rim trimming and PAO decovering the head. Fatigue fracture healed (black arrow) with substantially reduced pain

27-year-old female with osteogenesis imperfecta and bilateral protrusio (Fig. 11). She had increasing and constant pain from global pincer impingement in the right hip, while the left side remained nearly pain free. Radiographically the joint space on the right side was globally narrowed. The patient favored joint preservation after she had learned that the lifetime of THR with such bone quality is limited. Finally, she understood that joint preservation for her hip would be elaborate and that a good and lasting result cannot be guaranteed. The direction of reorientation being reversed to the classic correction would require a large rim trimming to start with. Surgical dislocation confirmed a reasonable cartilage layer of the femoral head with some osteophytes. Circumferential trimming, especially at the posteroinferior rim was combined with some osteochondroplasty at the head neck junction. While execution of the PAO cuts was easy, valgus correction of the acetabulum was rather difficult, mainly due to the osteoporotic bone. To bridge the large supraacetabular step as a result of the final position, a staircase-shaped plate had to be used. Healing of the osteotomy was uneventful; consolidation of the fatigue fracture could be observed at the 6-week control. At 2 years after surgery, the patient was pleased with the improved and pain-free ROM. Radiographic widening of the joint space was interpreted as sign of ongoing improvement.

### Case 6

**Fig. 12:**
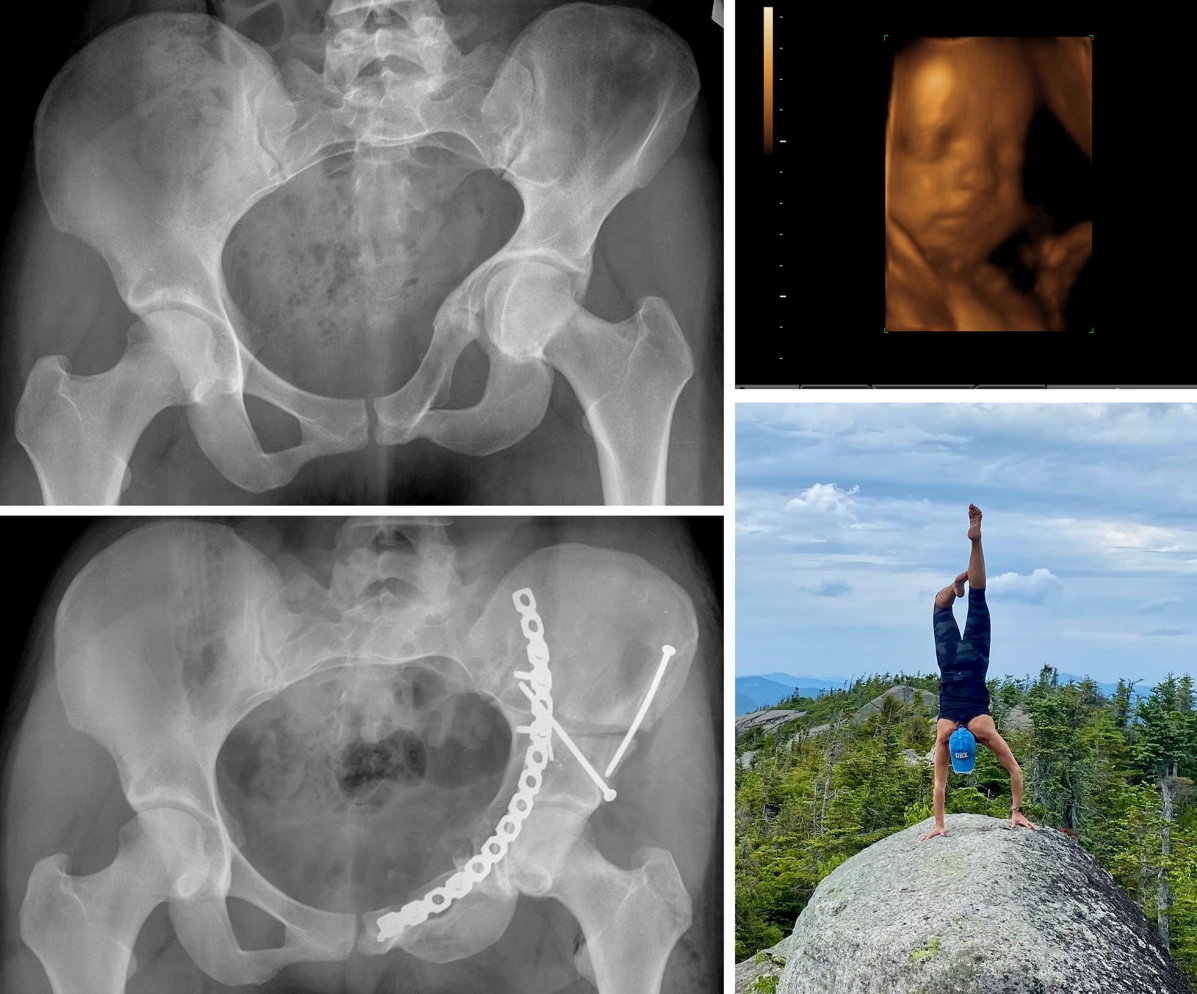
Posttraumatic protrusio observed in patient. **a** Posttraumatic protrusio and deformation of the hemipelvis in a 23-year-old female. Her gynecologist discussed the risk of abortion due to deviation of the uterus, which was the reason why she was asking for correction. A modified PAO was first tested on a plastic model. Correction of protrusio und pelvic deformation was assisted by a distractor. **b** The new position was stabilized with a long recoplate placed along the pelvic brim. Ultrasound was performed just before natural delivery. **c** At 16 years after surgery, she is now the mother of five children and is very active in outdoor activities. The straight leg is the operated one

A 23-year-old female with severe unilateral narrowing of the pelvic cavity after a conservatively treated complex periacetabular fracture in childhood (Fig. 12). The joint looked rather normal and hip pain or restricted motion were minimal; her problem was connected to the desperate desire to get pregnant and a statement of her gynecologist who expressed concerns whether the asymmetric narrowing could lead to a deviation of the increasing uterus with increasd risk of abortion. A three-dimensional (3D) model of the pelvis did not give a conclusive answer to the possibility of uterus deviation, but showed that the birth canal was somewhat narrow for a natural delivery. From an earlier case with pelvic deformation and successful use of a distractor, it seemed possible to use such a system for the necessary lateral and distal shifting of the medialized joint complex. Test surgery on a plastic model of the deformed pelvis revealed that a modified PAO, separating the posterior column proximally, would allow to lateralize the acetabular fragment sufficiently and recreate a normal curvature of the inner pelvic contour. For the verticalized pubic ramus an additional osteotomy near the symphysis was necessary. The best place for the Schanz’ screws of the distractor were the opposite iliac bone near the sacroiliac joint and at the ipsilateral femoral head. Best fixation was possible with a reconstruction plate placed along the pelvic brim. An ilio-inguinal approach would allow exposure of the entire hemipelvis. For the Schanz’ screw near the opposite sacroiliac joint, a short incision at the iliac crest would be sufficient. The three cuts around the joint and the additional cut of the pubis near the symphysis were executed as tested on the model. Manual traction did not dispalce the acetabular fragment much. Instrumented distraction, however, allowed slow but constant displacement while alignment could be assisted with instruments. The prebended plate was first fixed near the symphysis and on the mobile pubis segment. Proximal off-standing of the plate could be steadily converged to the stable iliac bone by alternate driving in the screws. This technique allowed an optimal final positioning of the acetabular fragment. Osteotomy healing was uneventful; shortly after she became pregnant. She finally had a natural delivery and did so 2 years after surgery with a second child. A total of 16 years after surgery, the patient, now a mother of 5 children, is pain free and likes outdoor activities very much.

### Case 7

**Fig. 13:**
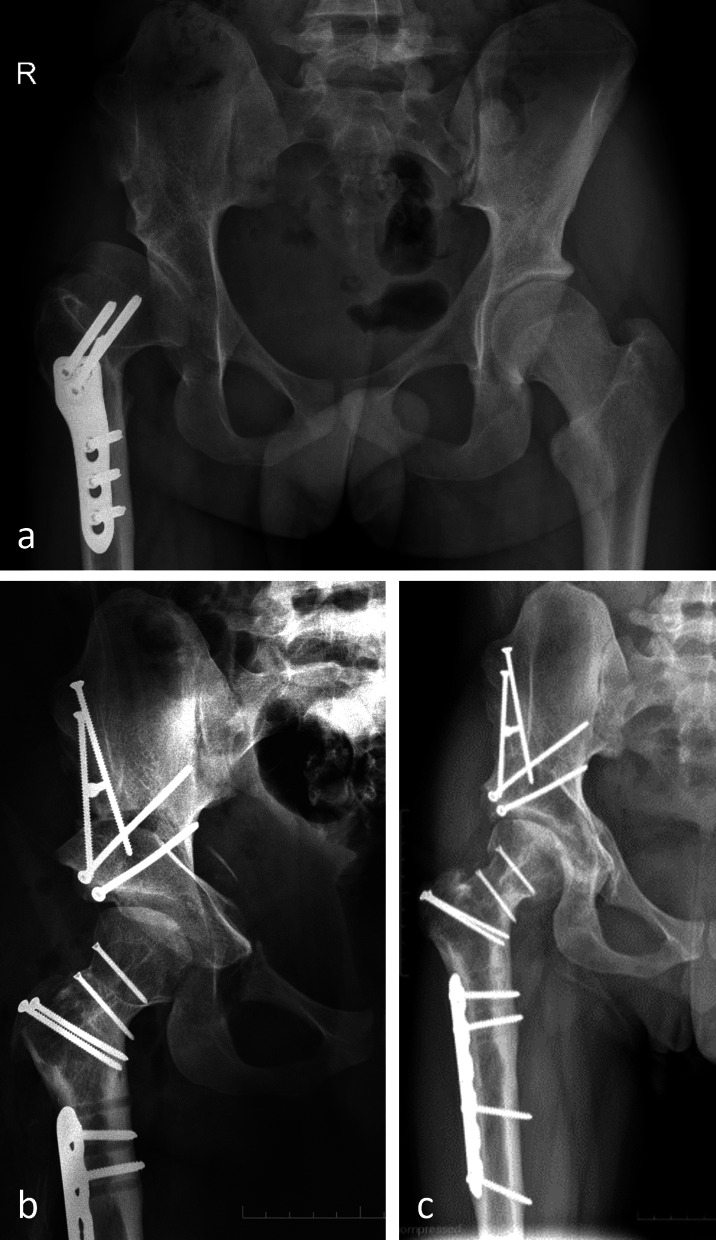
Severe deformity (courtesy Prof. P. Rego, Hospital da Luz, Lisbon, Portugal) of the right hip in a 16-year-old male after open reduction at the age of 1.5 years, followed by avascular necrosis. **a** Varus osteotomy at the age of 14 years, which did not improve limping and pain. **b** Postoperative radiography after complex surgery with relative lengthening of the neck, intracapital osteotomy to reduce the size of the head, and subtrochanteric derotation osteotomy followed by PAO. **c** The 4-year follow-up result with good and pain-free function

This case (Fig. 13) was operated by Professor Paulo Rego, a former fellow. It is presented to show that the techniques are learnable by others.

Complex deformity of the right hip in a 16-year-old male was observed. He had open reduction for DDH at the age of 1 year, which was followed by incomplete necrosis of the epiphysis. Together with ongoing subluxation, the acetabulum became shallow over a Perthes-like deformed femoral head. Pain and limping did not improve after intertrochanteric varus osteotomy at the age of 14 years. MRI showed a reasonable condition of the joint cartilage and computer simulation revealed an improved head sphericity with intracapital osteotomy [45]. The preoperative evaluation also exposed a high femoral neck anteversion. Surgery was started with the femoral side: the first ischial cut of the PAO was followed by relative neck lengthening and intracapital osteotomy and finally subtrochanteric derotation. The PAO was completed after turning the patient into supine position. The surgeon commented that the entire surgery was demanding and time consuming; however, the radiographic result was convincing. The 6-year follow-up radiography showed a result of correction close to a normal hip. The patient is pain-free, has a normal walking pattern and a nearly symmetrical ROM (courtesy Prof. P. Rego, Hospital da Luz, Lisbon, Portugal).

## Complications

Technical complications developed first and foremost during the learning curve, which in turn is dependent on the case load over time. In a literature review from 2006, in about 13 publications, the incidence of major complications ranged from 6% to 37% [46]. In our first series of 500 cases, the most frequent complication was severe under-, over-, and intraarticular correction counting for nearly 2% of complications [47]. Injury to one of the main nerves (femoral, sciatic, or obturator) follows, making up less than 1% of complications [48], a number that may have been influenced by strict observation of recommendations from a cadaver study about surgical circumstances leading to such injury [48]. In 1760 cases of the ANCHOR group, complication incidence was 2.1%, 50% (17 out of 36) of which were transient [49]. Neuromonitoring during PAO can identify the surgical maneuver endangering trauma to a nerve, but cannot per se prevent damage when the blow is heavy enough to cause a laceration [50].

## International adoption of the Bernese PAO

This is primarily the result of multiple publications coming from different centers over nearly 40 years since its introduction. Further promotion came from a series of instructional courses in different countries and from fellowships, mainly organized in Switzerland and the USA. Also, the ongoing mentorship for colleagues during their learning period should be remembered [51]. Switzerland has probably the highest concentration of orthopedic departments performing the Bernese periacetabular osteotomy, with most of the relevant publications coming from the Bernese Group [52]. Next are the USA and Canada, where several institutions work together in the ANCHOR group to study all aspects of surgery preserving the native hip joint [53]. One or more centers are practicing, and some do report about their results with the Bernese periacetabular osteotomy in Germany, England, Denmark, Italy, Spain, Portugal, Chile, Iran, and finally China, from where the most recent report was published [54].

## Future opportunities and challenges

The indication for (or against) PAO is still one of the most difficult decisions to be made. There is a tendency to perform the surgery even when the triradiate growth plate is still open, but little is known about how far one can go without risking surgery-related malformation after injuring the growth plate. Interestingly enough, deepening of the immature acetabulum for capsular arthroplasty does not seem to produce a malformation. Maybe molecular studies of the triradiate cartilage can give some ideas about its growth balance. The indication limit for older candidates is indirectly defined by the recognition that PAO results after the age of 40 years are statistically less favorable. However, maybe the direct reason could be the increase of cartilage degeneration with age. However, to use detailed information about the status of the joint cartilage a software would be required to quantify and locate the cartilage damage of femoral head and acetabulum seen on an MRI. In any case, today the Tönnis classification of hip joint arthrosis alone is not a good parameter for decision-making and follow-up conclusions.

While the geometry of the PAO cuts did not change, the approach already went through some modifications. Some represent a clear advantage; one showed a benefit together with only one minor handicap during cadaver testing. It would be helpful to have such or similar studies of comparison, including the preoperative expenditure for the minimally invasive techniques. Further propositions of computer assistance should be analyzed for expenditure versus impact.

Progress can be expected when this demanding surgery is concentrated in few centers with a high case load and when several such centers work together and collect their material in a central registry.

## Data Availability

The datasets used and/or analyzed during the current study are available from the corresponding author on reasonable request.

## Abbreviations

PAO: Periacetabular osteotomy
ASIS: Anterior superior iliac spine
AIIS: Anterior inferior iliac spine
